# The investigation of the volatile metabolites of lung cancer from the microenvironment of malignant pleural effusion

**DOI:** 10.1038/s41598-021-93032-y

**Published:** 2021-06-30

**Authors:** Ke-Cheng Chen, Shih-Wei Tsai, Xiang Zhang, Chian Zeng, Hsiao-Yu Yang

**Affiliations:** 1 Division of Thoracic Surgery, Department of Surgery, National Taiwan University Hospital, Taipei, Taiwan; 2 National Taiwan University College of Medicine, Taipei, Taiwan; 3 Institute of Environmental and Occupational Health Sciences, National Taiwan University College of Public Health, No. 17 Xuzhou Road, Taipei, 10055 Taiwan; 4 Department of Chemistry, University of Louisville, Louisville, KY USA; 5 Department of Public Health, National Taiwan University College of Public Health, Taipei, Taiwan; 6 Department of Environmental and Occupational Medicine, National Taiwan University Hospital, Taipei, Taiwan

**Keywords:** Cancer, Environmental sciences, Biomarkers

## Abstract

For malignant pleural effusions, pleural fluid cytology is a diagnostic method, but sensitivity is low. The pleural fluid contains metabolites directly released from cancer cells. The objective of this study was to diagnose lung cancer with malignant pleural effusion using the volatilomic profiling method. We recruited lung cancer patients with malignant pleural effusion and patients with nonmalignant diseases with pleural effusion as controls. We analyzed the headspace air of the pleural effusion by gas chromatography-mass spectrometry. We used partial least squares discriminant analysis (PLS-DA) to identify metabolites and the support vector machine (SVM) to establish the prediction model. We split data into a training set (80%) and a testing set (20%) to validate the accuracy. A total of 68 subjects were included in the final analysis. The PLS-DA showed high discrimination with an R^2^ of 0.95 and Q^2^ of 0.58. The accuracy of the SVM in the test set was 0.93 (95% CI 0.66, 0.998), the sensitivity was 83%, the specificity was 100%, and kappa was 0.85, and the area under the receiver operating characteristic curve was 0.96 (95% CI 0.86, 1.00). Volatile metabolites of pleural effusion might be used in patients with cytology-negative pleural effusion to rule out malignancy.

## Introduction

Lung cancer is the leading cause of cancer death worldwide, accounting for an estimated 1.80 million deaths in 2020^1^. More and more studies have attempted to identify specific metabolites, which can help study various metabolic pathways affected by tumors, thereby developing effective diagnostic and therapeutic strategies^2^. Among them, volatilome has attracted more attention in the metabolomics research of lung cancer. Volatilome contains all volatile organic compounds (VOCs) produced by changes in metabolic processes caused by disease^3^. VOCs are small molecular substances with low boiling points (less than 250 °C), which can be measured directly in the gas phase at room temperature, thus requiring minimum sample handling protocols^3^. Volatile metabolites produced during the physiological and pathological processes of lung diseases are released into the alveolar air^4^. The metabolites can also be directly involved in increasing cancer cell growth, driving glycolysis and tumor proliferation^5^.

Pleural effusions are a common manifestation of malignant and nonmalignant diseases. Malignant pleural effusion is a condition in which cancer causes an abnormal amount of fluid to collect between the thin layers of tissue (pleura) lining the outside of the lung and the wall of the chest cavity^6^. Lung cancer accounts for 36.0% of malignant pleural effusions, followed by breast (26%) and lymphoma (13.0%)^7^. Clinical factors predicting the diagnosis of malignant pleural effusions are symptoms lasting more than one month and the absence of fever^8^. Accurate pleural fluid analysis is critical to the correct staging of cancers and is of great significance to prognosis and treatment. For malignant pleural effusions, pleural fluid cytology is a diagnostic method for lung cancer, but its sensitivity is low (about 40–60%)^9^. Consequently, many patients need to undergo invasive diagnostic tests such as thoracoscopic pleural biopsy^10^.

**Figure 1:**
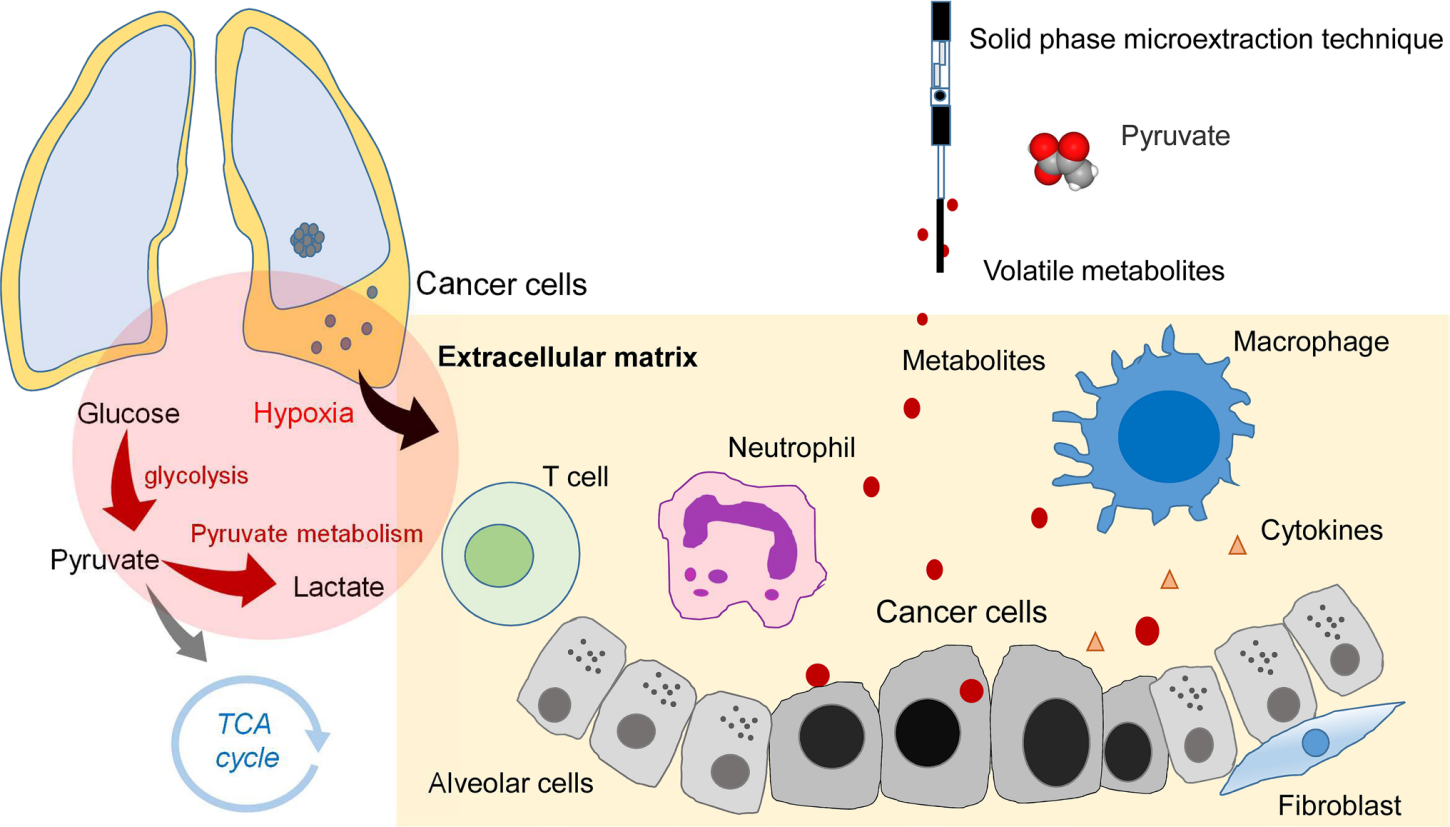
Schematic diagram showing volatilome in the microenvironment of pleural fluid of lung cancer. The hypoxic microenvironment of malignant pleural effusion increased glycolysis and generated volatile biomarkers of pyruvate.

The pleural space is an enclosed space between the visceral (lung) and parietal (chest wall) pleura. The tumor microenvironment in the pleural space is a complex network composed of tumor cells, fibroblast cells, inflammatory cells, and extracellular matrix^11^. The tumor microenvironment has now been recognized as a significant contributor to tumor progression and metastasis^12^. The pleural fluid originates from the lung interstitium and pleural capillaries^13^. In pathophysiology, the pleural effusion of lung cancer contains lung cancer cells, lymphocytes, and its metabolites^14^. The objective of this study was to diagnose lung cancer with malignant pleural effusion using the volatilomic profiling method (Fig. 1).

## Results

**Figure 2:**
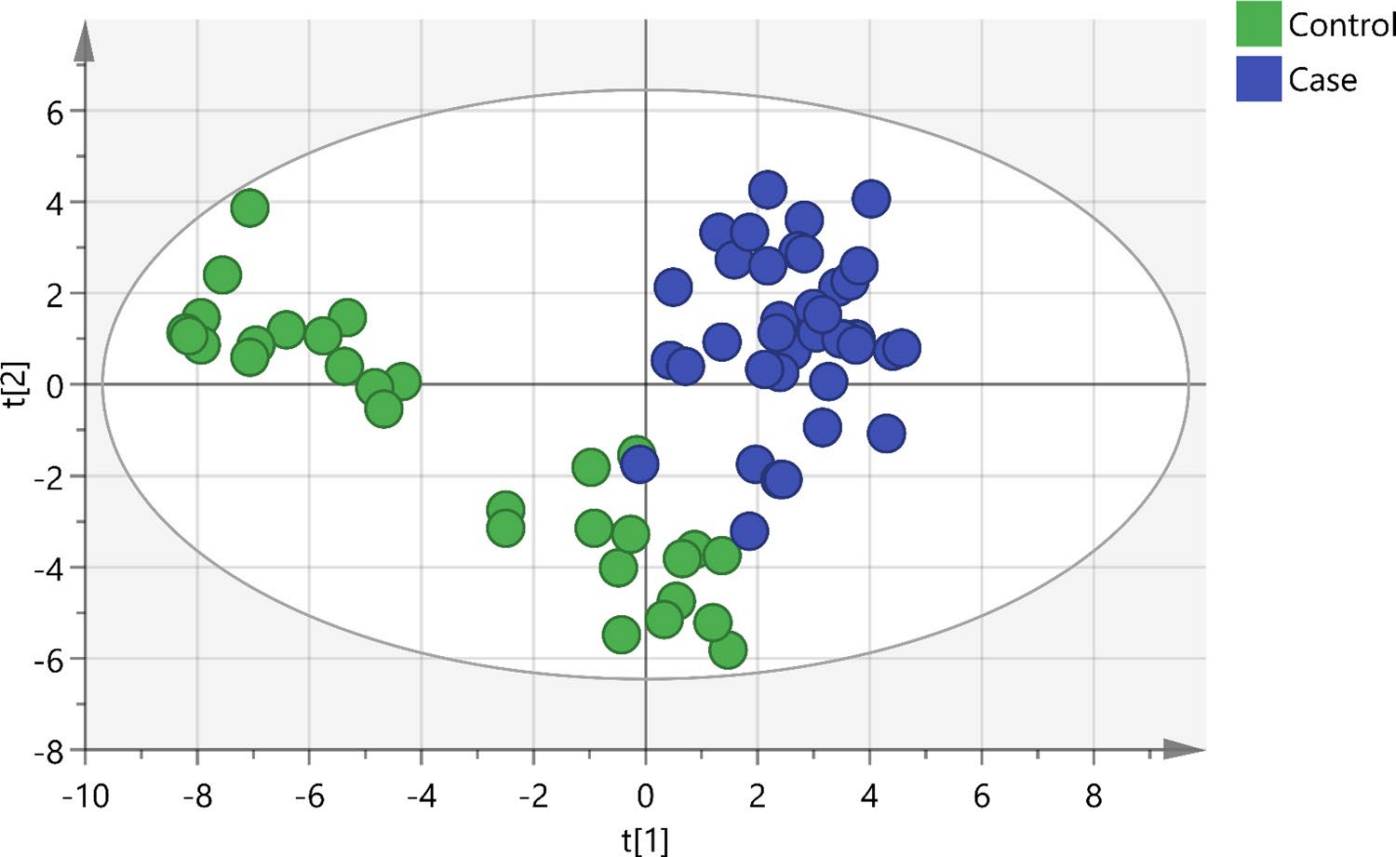
Scatterplot of scores obtained from all volatile metabolites by GC–MS of all samples. Blue plots show cases of lung cancer, and green plots show cases of nonmalignant disease as controls. The confidence ellipse based on Hotelling’s T^2^ test shows that there are no outliers. The score plot shows the excellent discrimination capability of the volatile metabolites of pleural fluid.

**Figure 3:**
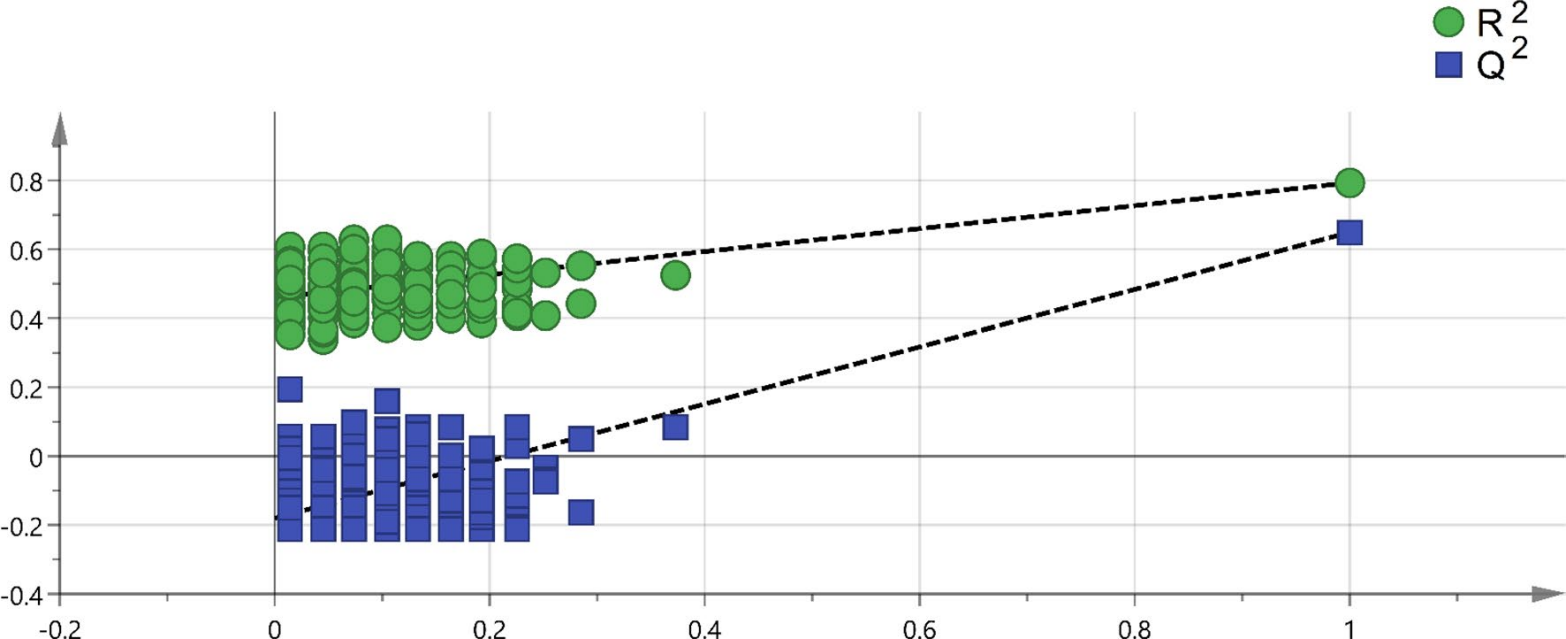
Permutation test of PLS-DA with VIP scores greater than 1. A permutation test with 200 random permutations and two components in the PLS-DA model showed R^2^ = 0.79 (green triangles) and Q^2^ = 0.65 (blue squares); values from the permuted test (bottom left) were significantly lower than the corresponding original values (top right).

**Figure 4:**
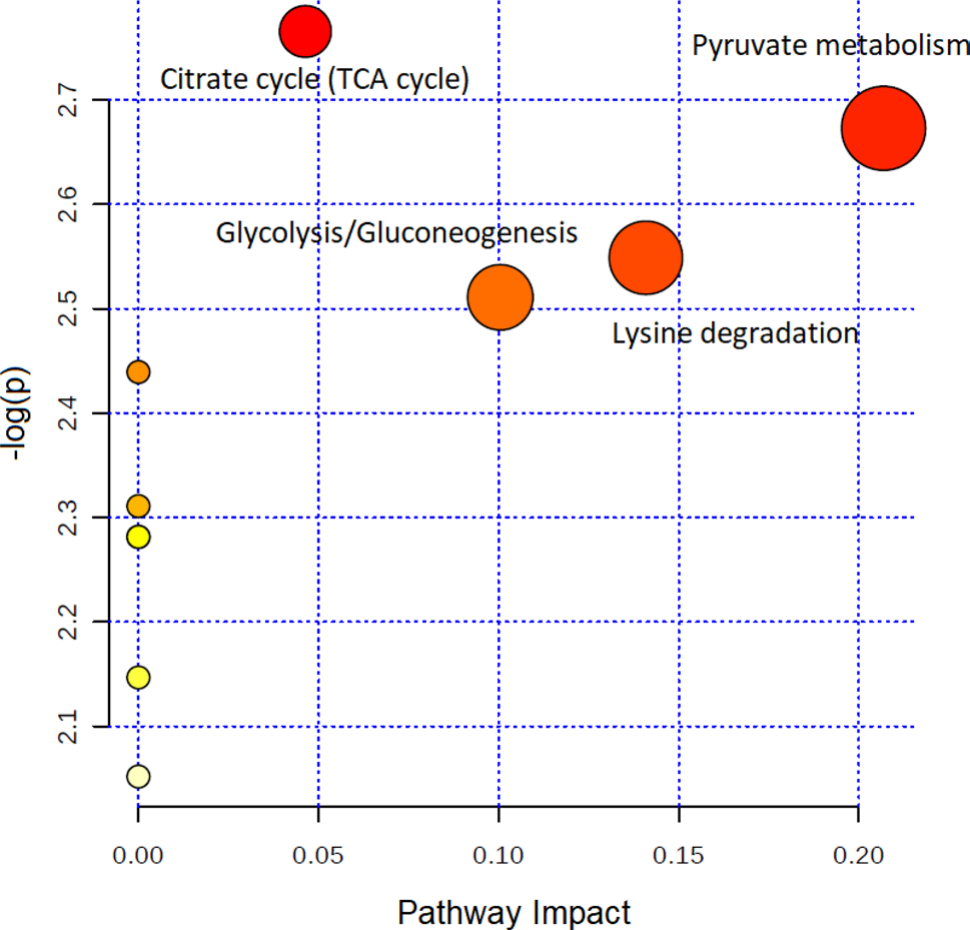
Topology-based pathway analysis showing metabolic pathways affected in lung cancer. The metabolome view shows matched pathways according to the *p* values from the pathway enrichment analysis and pathway impact values from the pathway topology analysis. The most impacted metabolic pathways are specified by the volume and color of the spheres (yellow, least relevant; red, most relevant) according to their statistical relevance *p* and impact values.

A total of 84 consecutive patients with pleural effusion were screened between April 23, 2018 and June 14, 2019. The case group included 43 lung cancers confirmed by pathological reports, and the control group included 41 patients with nonmalignant diseases, including pneumonia, heart failure, pneumothorax, inflammatory bowel disease, and Sjogren's syndrome. In the control group, 70.8% (17/24) was transudate, 20.8% was exudate (5/24), and 8.3% (2/24) did not have the protein and LDH data. In the case group, lung cancer patients with pleural effusion were in the advanced stage, and there was usually no further workup of pleural effusion. Among lung cancer patients undergoing diagnostic workup of pleural effusion, 63.6% (7/11) were exudate. In the control group, the cytology study confirmed that there were no malignant cells in the pleural fluid. We followed up subjects in the control group in April 2021, and none of them had cancer. The median follow-up period for the patients was 28 months. After excluding 18 subjects who had metastatic lung cancer caused by another type of cancer, renal failure with hemodialysis, diabetic ketoacidosis, lymphangioleiomyomatosis, or lung cancer combined with pneumonia or were currently smoking, 68 subjects were included in the final analysis. The majority of lung cancer patients were nonsmokers (71.1%), and the most common histological type was adenocarcinoma (94.7%) (Table 1). A total of 213 volatile metabolites were identified. The principal component analysis (PCA) score plot shows that the volatile metabolites from the malignant pleural effusion can discriminate between lung cancer patients and patients with nonmalignant diseases well (Fig. 2). The permutation test of partial least squares discriminant analysis (PLS-DA) yielded an R^2^ of 0.95 and a Q^2^ of 0.58. There were 78 metabolites whose variable importance on projection (VIP) scores were higher than 1. When we used the metabolites that showed VIP > 1 in PLS-DA, the permutation test showed an R^2^ of 0.79 and a Q^2^ of 0.65 (Fig. 3). PLS-DA also showed significant discrimination between lung cancer patients and patients with nonmalignant diseases (Figure S1). When we used all of the volatile metabolites of the malignant pleural effusion to establish a prediction model by support vector machine (SVM), the prediction accuracy in the test set was 0.93 (95% CI: 0.66, 0.998), the sensitivity was 83%, the specificity was 100%, and the kappa value was 0.85. The receiver operating characteristic curve (ROC) was 0.96 (95% CI 0.86, 1.00). The selected metabolites that were significantly different between the lung cancer patients and patients with nonmalignant disease as controls according to the bootstrapped Student's *t*-test with 1000 replications and VIP > 1 were summarised in Table 2. The ROC curves and boxplots of individual biomarkers were summarized in Figure S2. The pathway analysis revealed disturbances in pyruvate metabolism, the citric acid cycle (tricarboxylic acid cycle, TCA cycle), glycolysis, and lysine degradation (Fig. 4).

**Table 1:** Demographic characteristics of the study subjects with pleural effusion.

Characteristics	Lung cancer (n = 38)	Non-malignant control (n = 30)	*p* value
Age (yr), mean (SD)	65.7 (12.4)	77.5 (13.1)	0.00
Male, no. (%)	24 (63.2)	17 (56.7)	0.63
**Cigarette smoking**			
Pack-years, mean (SD)	41.3 (26.1)	29.4 (25.2)	0.34
Smoking status			0.60
Current smokers, no. (%)	0 (0.0)	0 (0.0)	
Former smokers, no. (%)	11 (28.9)	7 (23.3)	
Never smoked, no. (%)a	27 (71.1)	23 (76.7)	
Environmental tobacco smoke (%)	0 (0.0)	0 (0.0)	
**Tumour histological type**			
Squamous cell carcinoma, no. (%)	1 (2.6%)		
Adenocarcinoma, no. (%)	36 (94.7%)		
Small cell lung cancer, no. (%)	1 (2.6%)		
**Pleural effusion cytology exam**			
Positive for malignant cells	30 (78.9%)	0 (0.0%)	
Negative for malignant cells	8 (21.1%)	30 (100.0%)	
**EGFR mutation**			
Positive	18 (51.4%)	NA	
Negative	17 (48.6%)	NA

**Table 2:** Selected volatile metabolites with FC > 1.2 or < 0.8, VIP > 1, and *p* value by bootstrap *t-*test < 0.05.

Compound name	CAS number	Fold change	VIP	*p* value^#^
Cyclopropane, 1,1,2,2-tetramethyl-	4127-47-3	0.5	2.0	0.00
Oxirane, ethenyl-	930-22-3	1.6	1.9	0.00
3-Butene-1,2-diol, 1-(2-furanyl)-	19261-13-3	0.7	1.8	0.00
Methacrylic anhydride	760-93-0	0.6	1.8	0.00
2-Pentanone, 4-amino-4-methyl-	625-04-7	1.4	1.8	0.00
Cyclohexane, 1-methyl-2-propyl-	4291-79-6	1.4	1.6	0.00
2-Ethylthiolane, S,S-dioxide	10178-59-3	1.4	1.5	0.00
Hexanenitrile, 5-methyl-	19424-34-1	1.3	1.3	0.01
Acetic acid ethenyl ester	108-05-4	1.3	1.3	0.01
1-Butene, 2,3-dimethyl-	563-78-0	0.7	1.3	0.02
2,3-Butanedione	431-03-8	0.7	1.4	0.02
2-Chloroaniline-5-sulfonic acid	98-36-2	1.3	1.3	0.02
3-Butene-1,2-diol	497-06-3	0.7	1.2	0.02
Methyl vinyl ketone	78-94-4	1.4	1.2	0.03
Silane, tetramethyl-	75-76-3	1.4	1.2	0.04
Cyclotetrasiloxane, octamethyl-	556-67-2	1.3	1.1	0.04

^#^*p* value of bootstrapped Student's *t*-test with 1000 replications.

When we applied classical ROC-based biomarker analyses for lung cancer, the volatile tumor markers with the ROC > 0.75 included trimethyl[4-(1,1,3,3,-tetramethylbutyl)phenoxy]silane (CAS No. 78721-87-6), acetic acid, trifluoro-, 1-methylethenyl ester (CAS No. 400-39-5), oxirane, ethenyl- (CAS No. 930-22-3), benzaldehyde, 4-methoxy-3-(3-methyl-4-nitrophenoxymethyl)- (CAS No. 329222-76-6), 4-amino-4-methyl-2-pentanone (CAS No. 625-04-7), 1-methyl-2-propyl-cyclohexane (CAS No. 4291-79-6), and 2-Ethylthiolane, S,S-dioxide (CAS No. 10178-59-3) (Figure S2). When we used FC and the bootstrapped *t*-test to select important volatile tumor markers. We found that the branched-chain alkane 1-methyl-2-propyl-cyclohexane (fold change (FC) = 1.39, *p* value = 0.00) is an important volatile biomarker of lung cancer. We also noted that some ketones were significantly increased in lung cancer subjects, including methyl vinyl ketone (FC = 1.37, *p* value = 0.03) and 4-amino-4-methyl-2-pentanone (FC = 1.40, *p* value = 0.00).

**Figure 5:**
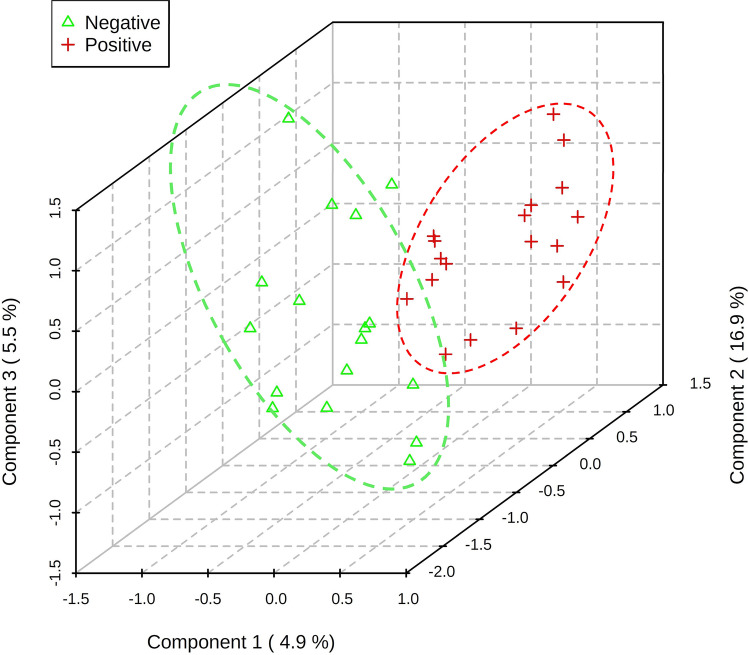
The 3D score plot shows a clear distinction in VOC between lung cancer patients with and without EGFR mutations. The red plus symbols indicate lung cancer patients with EGFR mutation. The green triangle symbols indicate lung cancer patients without EGFR mutation. The explained variances are shown in brackets.

We applied the PLS-DA to distinguish the volatile metabolites between lung cancer patients with and without EGFR mutation. The volatile metabolites can be separated well by volatile metabolites (Fig. 5). When we applied classical ROC-based biomarker analyses, the volatile tumor markers with the ROC > 0.75 included butanoic acid, (tetrahydro-2-furanyl)methyl ester (HMDB ID HMDB0036188), 1-hexene, 3,4-dimethyl- (CAS No. 16745-94-1), and 2-undecen-4-ol (CAS No. 22381-86-8). When we used FC and the bootstrapped *t*-test to select important volatile tumor markers. We found that the 2-undecen-4-ol (FC = 1.51, *p* value = 0.01), 2H-tetrazole, 2-methyl- (FC = 1.37, *p* value = 0.03), 2-propanol, 1-chloro-3-propoxy- (FC = 1.47, *p* value = 0.04) were significantly increased in the lung cancer subjects with EGFR mutation. The ethyl [5-hydroxy-1-(6-methoxy-4-methyl-3-quinolinyl)-3-methyl-1H-pyrazol-4-yl]acetate (FC = 1.24, *p* value = 0.04), cyclobutylamine (FC = 0.74, *p* value = 0.01), butanoic acid, (tetrahydro-2-furanyl)methyl ester (FC = 0.72, *p* value = 0.01), hexane, 2,3,5-trimethyl- (FC = 0.70, *p* value = 0.02), 1H-Tetrazole-1-ethanol (FC = 0.78, *p* value = 0.04), cyclopropene (FC = 0.74, *p* value = 0.04), 4H-1,2,4-Triazol-4-amine (FC = 0.75, *p* value = 0.045), and allyl acetate (FC = 0.75, *p* value = 0.046) were significantly decreased in the lung cancer subjects without EGFR mutation.

## Discussion

To the best of our knowledge, this is the first study to explore the volatilome of lung cancer in the pleural fluid. The volatilome identified from the pleural microenvironment can reflect the altered metabolomic changes of existing lung cancer. The analysis of volatile metabolites from malignant pleural effusion has high discrimination accuracy for lung cancer and EGFR mutation.

This study showed that the volatile metabolites identified from malignant pleural effusion of lung cancer were primarily methylated alkanes. The findings are consistent with previous studies that also showed that alkanes (hydrocarbons), methylated alkanes, and branched-chain alkenes are commonly reported as potential volatile tumor markers of lung cancer^15,16^. Alkanes and methylated alkanes have been reported to be the end-products of lipid peroxidation in endogenous biochemical pathways^15^. Oxidative stress plays an important role in the pathogenesis of lung cancer, as it increases the generation of reactive oxygen species (ROS), which will cause DNA damage and then result in lung cancer^17^. Ketone production is associated with stress, such as cancer, where increased fatty acid oxidation leads to the formation of ketone bodies. Moreover, increased protein metabolism, such as during cancer-induced cachexia, can increase the generation of ketones in the body^18^. Because volatile tumor markers with missing values in more than 75% of the samples were deleted during data preprocessing, some potential metabolites might be underreported. We also compared the detection rate for all volatile tumor markers between lung cancer patients and controls by Fisher’s exact test to select important volatile tumor markers. A total of 41 metabolites showed statistical significance by Fisher’s exact test (Table S1). Among them, the alkyl aldehyde hexanal has been reported to have a significantly higher concentration in the exhaled breath of lung cancer patients than in that of smokers and healthy controls^19^. Liu et al.^20^ used GC–MS to analyze the headspace air of pleural effusion samples and reported that cyclohexanone, 2-ethyl-1-hexanol, and 1,2,4,5-tetramethyl benzene were volatile tumor markers of lung cancer. In this study, we did not obtain similar findings. Moreover, 1,2,4,5-tetramethyl benzene might come from exogenous sources, including tobacco and environmental pollution. We suggest that further studies include a targeted analysis to validate these volatile tumor markers.

Our pathway topology analysis identified volatile metabolites involved in pyruvate metabolism, citric acid cycle (TCA cycle), glycolysis, and lysine degradation. These metabolic pathways play an essential role in cancer biology^21^. Due to rapid proliferation, cancer cells have increased anabolic metabolism and energy demands. The hypoxic microenvironment activates glycolysis, and the majority of pyruvate is converted into lactate^22^. Fan et al. used ^13^C-isotopomer-based metabolomic analysis to analyze the metabolic perturbation in lung cancer patients. The results showed that the activation of glycolysis and the TCA cycle in human lung tumors^23^. Musharraf et al. used GC–MS to identify the comparative and distinguishing metabolite patterns for lung cancer from serum. The pathway analysis also revealed disturbances in pyruvate metabolism and the TCA cycle^24^. Lysine degradation was associated with cancer cell proliferation. Activation of the lysine degradation pathway impairs cancer cell proliferation^25^. There are few volatile metabolites in the human metabolome database (HMDB) and the KEGG database^26^, and the metabolite included in the metabolomic pathway is limited. The reason may be that the primary type of pleural effusion in the control group was transudate, so there were fewer metabolites. However, we suggest more studies to enrich the volatile metabolites in these databases and facilitate further research to explore the volatilome of diseases. As the tumor microenvironment is essential to understand and therapeutically target cancer cell metabolism^27^, the impact of tumor microenvironment on cancer progression is not well understood^28^. We suggest further studies can further determine the alterations of pyruvate metabolism and survival of lung cancer.

Metabolomic analyses can be classified as targeted or untargeted. Targeted analysis measures selected compounds known as metabolites of specified biological or pathological pathways, and this method involves the use of standard solutions of these compounds for analysis^29^. In contrast to targeted metabolomic analysis, untargeted analysis scans all ions within a specific mass range to explore novel metabolites without standard solutions^30^. In an untargeted metabolomic analysis, the peaks of volatile tumor markers in the total ion chromatograms (TICs) obtained by GC–MS analysis are often overlapped by matrix peaks and are difficult to distinguish from noise^31^. Data preprocessing is necessary for untargeted analysis^32^. In this study, we used the online software MZmine for data preprocessing. The software supports several steps of data preprocessing, including mass detection, chromatogram construction, deconvolution, alignment, and gap-filling^33^. In our analysis, we used the gap-filling method. When the percentage of ions detected for all samples was > 60%, the missing values were filled by the gap-filling method. We carefully examined the raw chromatographic data with experts and noted that gap-filling would result in the misidentification of ions. Thus, we decided not to use the gap-filling method in our final analysis. Gap-filling remains a significant challenge that might result in uncertainty in the gap-filled values^34^. We suggest that further studies carefully examine the results of gap-filling to prevent the false discovery of metabolites. According to the eighth edition of TNM staging, a lung cancer patient with pleural effusion is consider M1a thus stage 4, these metabolites identified in the advanced staged patients might not be suitable for early screening for lung cancer. To increase the numbers of identified VOCs, future research can apply two-dimensional gas chromatography using a time-of-flight mass spectrometric detector (GCxGC-TOFMS) to analyze the VOCs.

### Strengths and limitations

The strength of this volatilomic study is to analyze the volatile metabolites in the microenvironment of pleural space to prevent contamination of ambient air during the exhalation collection procedure. The analysis of VOCs in exhaled breath has been applied in lung cancer^35^. However, the analysis of VOCs from exhaled breath might be affected by the expiratory flow rate, breath-holding, the oral cavity, diet, and the anatomical dead space of the upper airway^36^. This study found a reliable source to analyze the volatile metabolites of lung cancer, which can prevent the false discovery of volatile metabolites.

There are still limitations. To extract volatile, low-molecular-mass, and polar analytes, we selected a Carboxen/Polydimethylsiloxane (CAR/PDMS)-coated fiber following a previous study that also analyzed the volatile tumor markers of pleural effusion^20^, and the results show that the extracted volatile metabolites have high diagnostic accuracy. However, CAR/PDMS is most suitable for the molecular weight range of 30–225, and macromolecular esters and amino acids outside that range would not be detected. Therefore, the selectivity of solid-phase microextraction (SPME) may lead to the loss of potential volatile tumor markers consisting of esters and amino acids. We suggest that further research uses a less selective preprocessing approach to explore a broader range of potential volatile tumor markers.

## Conclusions

Malignant pleural effusion is a microenvironment that contains lung cancer cells, lymphocytes, and their metabolites. Analysis of metabolites from pleural space can identify metabolites involved in the proliferation of lung cancer. This is the first study to explore the volatilome of lung cancer in the pleural microenvironment. Our results showed that the volatile metabolites identified from malignant pleural effusion of lung cancer were primarily methylated alkanes. We suggest that the analysis of volatile metabolites of pleural effusion might be used in patients with cytology-negative pleural effusion to rule out malignancy and reduce the need for thoracoscopic pleural biopsy.

## Methods

### Subjects and clinical data

We conducted a case–control study at National Taiwan University Hospital. We recruited lung cancer patients with malignant pleural effusion and patients with pleural effusion without malignancy who underwent thoracentesis as the control group. The eligibility criteria of the lung cancer patients were primary lung cancer with pleural effusion that was ascertained by physicians and confirmed based on pathological reports and medical history. The control group was collected by incidence sampling. All methods were carried out following relevant guidelines and regulations. The ethics committee of the National Taiwan University Hospital approved the research protocol (No. 201803028RINC). All subjects provided written informed consent before the study.

### Exclusion criteria

Pregnant women and young people less than 20 years old were also excluded from enrollment. We excluded subjects with metastatic lung cancer, other types of cancer, renal failure with hemodialysis, diabetic ketoacidosis, and current smokers that may influence metabolisms in the final analysis^4^.

### Medical, occupational and environmental history

We obtained a medical history from medical records that included information regarding the tumor stage, medication, imaging findings, serum lactate dehydrogenase, glucose, total protein, white blood cell, blood urea nitrogen, creatinine, alanine aminotransferase levels, pleural fluid LDH, total protein, glucose, white blood cell, red blood cell levels, malignant pleural effusion cytology findings, pathology findings, and EGFR mutation. A face-to-face interview was carried out to obtain a detailed occupational history, which included the year occupation started and ended, the cumulative number of years for each occupation, and the tasks involved in each type of occupation. Because cigarette smoking may be a confounding factor, the history of cigarette smoking and environmental tobacco smoke exposure was obtained. The study obtained lifestyle factors that included habitual cooking at home, habitual indoor burning incense, and habitual use of essential oil (defined as more than three times per week).

### Ultrasonic cleaning

All procedures were performed in a closed system to prevent contamination by environmental air. We rinsed a glass vial with acetone and then washed it with deionized distilled water (ddH_2_O) three times, followed by soaking the vial in ddH_2_O and sonicating it for 15 min in a ddH_2_O bath three times.

### Sample collection and preparation

Physicians performed thoracentesis and drainage pleural effusion. We collected the pleural effusion from the sterile bottle with a gas-tight syringe (SGE Syringes, Trajan, Victoria, Australia). We transferred the fluid to a 10-mL vacutainer tube without anticoagulant (BD Vacutainer Plus Plastic Serum Tubes, Becton Dickinson, Franklin Lakes, NJ, USA) to prevent contamination. The tubes were stored in a refrigerator to keep the temperature at 4 °C before centrifugation. The collected samples were sent to the laboratory and centrifuged within three hours. The pleural fluid was centrifuged at 1500× *g* for 10 min by a refrigerated centrifuge at 4 °C, designed for heat-sensitive samples (Centrifuge 5702R, Eppendorf, Hamburg, Germany). The supernatant was transferred into a new vacutainer without anticoagulant and then stored at − 80 °C until further analysis. To prevent contamination by environmental air, all procedures were performed in a closed system. We placed a stir bar into a 4-mL glass vial, sealed it with a Teflon/silicone septum, and then filled it with nitrogen. The pleural fluid samples were first thawed at 4 °C. Then, we used a gas-tight syringe to inject 2 mL of pleural fluid into the sealed 4-mL glass vial (Figure S3).

### Volatilome analyses

We analyzed the headspace air of the pleural effusion with an untargeted chromatography-mass spectrometry (GC–MS) analysis and SPME technique to analyze the volatile organic compounds of the pleural fluid. The method followed a study reporting the investigation of volatile organic metabolites in lung cancer pleural effusion with the extraction time, desorbed time, and mass range modified based on our pilot study^20^. The GC–MS analysis was performed on a Hewlett–Packard 6890 GC system equipped with a 5973 mass-selective detector (Agilent Technologies, Santa Clara, CA, USA) and a DB-5 MS column 30 m × 0.25 mm (i.d.) in size with a film thickness of 0.25 μm (J&W Scientific, Folsom, CA, USA). Based on a suggested SPME method^37^, we chose a 75-μm carboxen/polydimethylsiloxane (CAR/PDMS) SPME fiber (Supelco, Bellefonte, PA, USA) that is suitable for the extraction of volatile, low­molecular-mass and polar analytes^37^. Before analyzing any samples, we used bromofluorobenzene as an external standard for instrument performance and ran the fiber blank to ensure no contamination of the GC–MS analysis.

The SPME fiber was inserted into the headspace of the 4-mL vial and exposed for 25 min at 50 °C in an oil bath under stirring at 800 rpm. After extraction, the fiber was inserted into the GC injector for analysis. The adsorbed compounds on the fiber were desorbed at 250 °C in the GC injector for 10 min. Then, the thermally desorbed trace components were separated by a capillary column with helium flow at a rate of 1.3 mL/min using the splitless mode. The chromatographic analytical column temperature was initially set at 35 °C with a 1-min hold and then programmed up to 230 °C at a rate of 10 °C/min. The line transfer temperature was 230 °C. For the MS measurement, ionization was executed by the electron impact (EI) method at 70 eV. We analyzed the VOCs by MS in full scan mode from 33 to 300 m/z.

### Statistical analysis

We applied heatmaps and PCA for data visualization. The normalized and logarithm-transformed GC–MS data were used for PLS-DA. In PLS-DA, we calculated the VIP for each component and obtained an average value. We used R^2^ to evaluate the fit of the model, Q^2^ to assess the predictability of the model, and FC to show the importance of each metabolite. FC is a quantitative measure for changes in metabolite concentrations relative to a reference group^38^. A larger absolute value of FC indicates a more significant difference in the average peak area (metabolite intensity) between lung cancer patients and patients with nonmalignant disease as controls. We used a bootstrapped Student’s *t*-test with 1000 replications to compare the mean values between these two groups. We also used SVM with the polynomial kernel to establish a prediction model for lung cancer with all identified metabolites. To validate the model, we randomly split data into a training set (80%) for model derivation and a test set (20%). We determined the accuracy, kappa, and area under the ROC in the test set. We also conducted a KEGG metabolic pathway analysis using metabolites identified by the online software MetaboAnalyst and the Kyoto Encyclopedia of Genes and Genomes (KEGG) database, and VIP > 1^39^. All statistical analyses were conducted using R 3.6.1 software, SIMCA 14 (Umetrics, Malmo, Sweden), and IBM SPSS Statistics (version 20).

### Sample size estimation

We calculated the sample size by estimating the standard error of the percentage of correctly classified patients^40^:

2 $${\text{SE}} = ~\sqrt {\frac{{C\left( {1 - C} \right)}}{n}}$$

where SE is the standard error, *C* is the percentage of patients classified correctly, and *n* is the estimated sample size. Based on our previous study that used an electronic nose to analyze the volatile metabolites in exhaled breath to diagnose lung cancer, the accuracy was 0.90 (95% CI = 0.80–0.99)^35^. We use the SE of 0.05 and the acceptable accuracy (*C*) of 0.8. The required sample size is 64.

## Supplementary Information

Supplementary Information.

## Data Availability

All the experimental procedures are publicly available in *Protocols.io* (https://www.protocols.io/view/untargeted-analysis-of-pleural-effusion-of-lung-ca-6xthfnn).
