# Supplementary material for: The investigation of the volatile metabolites of lung cancer from the microenvironment of malignant pleural effusion
Source: Sci Rep. 2021 Jun 30;11:13585. doi: 10.1038/s41598-021-93032-y (PMC8245642; doi:10.1038/s41598-021-93032-y)
Supplement: Supplementary file 1 — Supplementary Information. [file 41598_2021_93032_MOESM1_ESM.pdf]

Title: The investigation of the volatile metabolites of lung cancer from the microenvironment of malignant pleural effusion

Authors: Ke-Cheng Chen,[a,b] Shih-Wei Tsai,[c] Xiang Zhang,[d] Chian Zeng,[c] Hsiao-Yu Yang,[c,e,f]*

Affiliation:

[a] Division of Thoracic Surgery, Department of Surgery, National Taiwan University Hospital, Taipei, Taiwan

[b] National Taiwan University College of Medicine, Taipei, Taiwan

[c] Institute of Environmental and Occupational Health Sciences, National Taiwan University College of Public Health, Taipei, Taiwan

[d] Department of Chemistry, University of Louisville, Louisville, Kentucky, USA

[e] Department of Public Health, National Taiwan University College of Public Health, Taipei, Taiwan

[f] Department of Environmental and Occupational Medicine, National Taiwan University Hospital, Taipei, Taiwan

* Correspondence: hyang@ntu.edu.tw; No. 17 Xuzhou Road, Taipei, Taiwan 10055.

Tel.: +886-233668102

Data preprocessing

Preprocessing of the raw GC-MS data followed a standardized protocol that included mass detection, chromatogram construction, deconvolution, and alignment. All procedures were performed using the open-source software MZmine 2 (version 2.32), which has been widely used in many metabolomic studies[1]. The parameters used in MZmine 2 were in accordance with the protocol reported by Hayashi et al. and Jiang et al. for untargeted GC-MS analysis [2,3] with the minimum time span modified according to our pilot study (Table S1). The compounds were identified using the National Institute of Standards and Technology (NIST) library of the NIST 11 database (NIST/EPA/NIH Mass Spectral Library, 2011 version), and the minimum value for match factor was set as 600 (maximum spectrum similarity score, 1000). The ion at a given retention time with the highest average peak area was considered a metabolite.

The GC-MS data were preprocessed according to a validated procedure reported by Niu et al. [4] using the web-based tool MetaboAnalyst (http://www.metaboanalyst.ca) [5]. The preprocessing procedures are listed in the supplementary materials. We used ddH_2O as the method blank sample and calculated the relative standard deviation (RSD) of the method blank to assess the reproducibility of the measurements. The RSD of the three measurements was 0.5.

The preprocessing procedures of GC-MS data includes:

Step 1. Removal of unreliable values: a variable was kept if the variable had a nonzero value for at

least one out of four replicates in each of the lung cancer patients and patients with nonmalignant diseases.

Step 2. Treatment of zeros: zeros remaining after the removal of unreliable values were replaced by the minimum value in the dataset divided by 2.

Step 3. Logarithm transformation: the generalized logarithm (glog) is a simple variation of the ordinary logarithm to address zero or negative values in the data set. Its formula is shown below:

$$\text{glog}_2(\text{x}) = log_2 \frac{x + \sqrt{x^2 + a^2}}{2} \quad (1)$$

Where a is a constant with a default value of 1.

Step 4. Normalization: normalization by a reference sample, also known as probabilistic quotient normalization, is a robust method to account for different dilution effects of biofluids. This method is based on the calculation of the most probable dilution factor (median) by examining the distribution of the quotients of the amplitudes of a test spectrum by those of a reference spectrum.

Table S1. Comparison of metabolites between lung cancer patients and nonmalignant controls by Fisher's exact test

**Compound name**	***p*-value**
3(2H)-Benzofuranone, 6-methoxy-2-[(3-methoxyphenyl)methylene]-, (E)-	0.00
2-Propenoic acid, 1-methylpropyl ester	0.00
Propanesulfonylacetonitrile	0.00
Ethanedioic acid, bis(trimethylsilyl) ester	0.00
2-Phenyl-3-methyl-pyrrolo(2,3-b)pyrazine	0.00
3-Heptanone, 5-methylene-	0.00
3-Butene-1,2-diol, 1-(2-furanyl)-	0.00
Isobutane	0.00
1-Pentene, 3-ethyl-2-methyl-	0.00
2-t-Butyl-5-(dimethoxy-phosphoryl)-3-methyl-4-oxoimidazolidine-1-carboxylic acid, t-butyl ester	0.00
3,5-Dimethyl-1,6-heptadien-4-ol	0.00
Butylamine, N-acetyl-1-cyano-2-ethyl-	0.00
Vinyl crotonate	0.00
Piperoxan	0.00

(2S,3S)-(-)-3-Propyloxiranemethanol	0.01
1,3-Dioxolane, 2-pentadecyl-	0.01
2-Propyn-1-ol, acetate	0.01
Cyclohexane, 1-bromo-4-methyl-	0.01
Oxetane, 3-(1-methylethyl)-	0.01
Sebacic acid, nonyl 2-phenylphenyl ester	0.01
Borane, trimethyl-	0.01
(2R,4aS,5S,8aS)-2,5-Dipentyldecahydroquinoline	0.01
3-Methylpentan-3-yl 2-methylbutanoate	0.01
Pentane, 2-chloro-4-methyl-	0.01
Trimethylsilyl dipropylphosphinate	0.01
2-Propenoic acid, ethenyl ester	0.02
1-(tert-Butyl)-3-methyl-piperidine	0.03
1,2,4,5-Tetrazine, 3,6-dimethyl-	0.03
2-Methylmalonodiamide	0.03
2-Propanone, 1,1,3,3-tetrachloro-	0.03
Benzene, 1,1'-(1,1,10,10-tetramethyl-1,10-decanediyl)bis[3,4-dimethyl-	0.03
Cyanamide	0.03
Hexane, 3-methyl-4-methylene-	0.03

Uracil, 1,3,6-trimethyl-5-(3-methyl-1H-indol-2-yl)-	0.03
6H-Benzofuro[3,2-c][1]benzopyran, 3,9-dimethoxy-	0.04
Benzenamine, 4-bromo-2-chloro-	0.04
Formic acid, ethenyl ester	0.04
Oxiranemethanol, (R)-	0.04
Hex-1-en-3-ol, 1,1-dibromo-	0.05
Hexanal	0.05
p-Xylene	0.05

Table S2. The parameters of MZmine

**Step**	**Parameter**	**Value**
Mass detection		
	Mass detection,	Centroid
	Noise level (positive ionization mode)	50
	Mass detection, MS level	1
Chromatogram builder		
	Min time span (min)	0.04
	Min height	2.5E2
	m/z tolerance	0.5 m/z or 100 ppm
Chromatogram deconvolution		
	Algorithm	Savitzky–Golay
	Min peak height	2.5E2
	Min peak duration	0 – 10 min
Alignment		
	Algorithm	Join aligner
	m/z tolerance	0.5 m/z or 100 ppm
	Weight for m/z	1
	Retention time tolerance	0.05 absolute (min)
	Weight for RT	1
Gap filler		
	Algorithm	Same m/z and RT range gap filler
	m/z tolerance	0.5 m/z or 100 ppm
NIST MS Search		
	Ionization method	No ionization
	Retention time tolerance	0.05 absolute (min)
	Max. peak per spectrum	10
	Min. match factor	600
	Min. reverse match factor	0

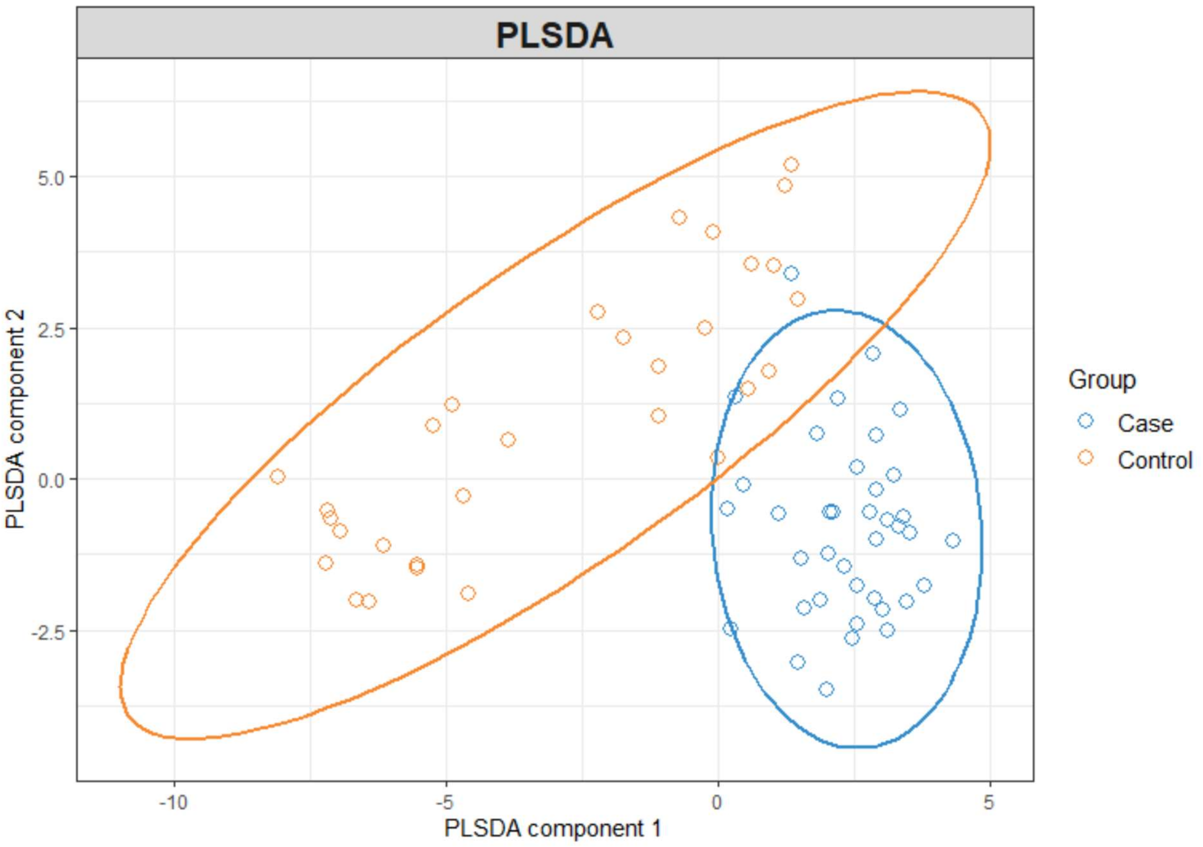

Figure S1. PLS-DA score plot. The blue circles indicate lung cancer cases, and the orange circles indicate nonmalignant controls. We included 78 metabolites with VIP > 1 in the PLS-DA. The score plot shows that lung cancer patients and nonmalignant controls can be distinguished well.

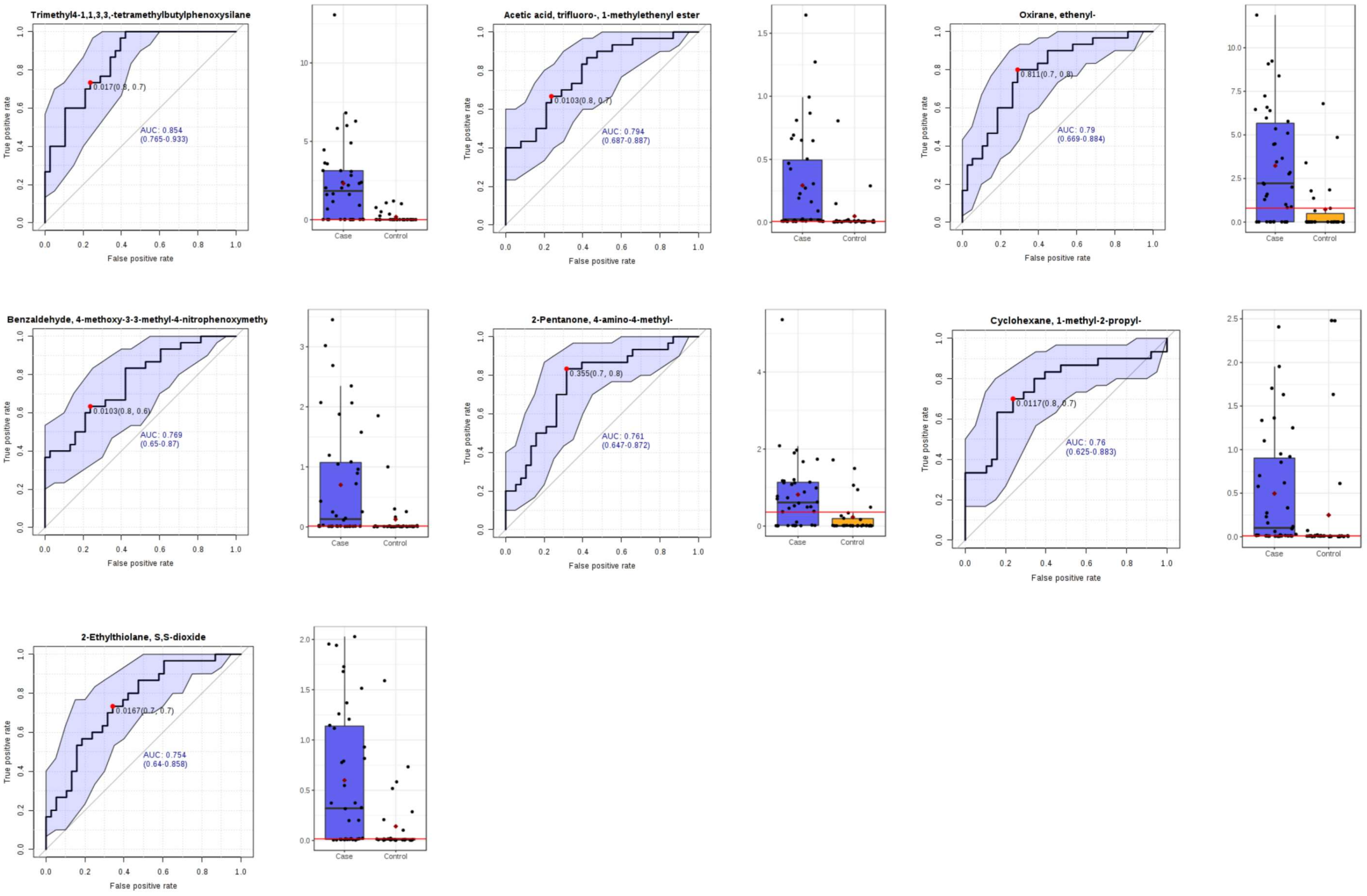
Trimethyl4-1,1,3,3,-tetramethylbutylphenoxysilane
0.017(0.8, 0.7)
AUC: 0.854
(0.765-0.933)
Acetic acid, trifluoro-, 1-methylethenyl ester
0.0103(0.8, 0.7)
AUC: 0.794
(0.687-0.887)
Oxirane, ethenyl-
0.811(0.7, 0.8)
AUC: 0.79
(0.669-0.884)
Benzaldehyde, 4-methoxy-3-3-methyl-4-nitrophenoxymethy
0.0103(0.8, 0.6)
AUC: 0.769
(0.65-0.87)
2-Pentanone, 4-amino-4-methyl-
0.355(0.7, 0.8)
AUC: 0.761
(0.647-0.872)
Cyclohexane, 1-methyl-2-propyl-
0.0117(0.8, 0.7)
AUC: 0.76
(0.625-0.883)
2-Ethylthiolane, S,S-dioxide
0.0167(0.7, 0.7)
AUC: 0.754
(0.64-0.858)
True positive rate
False positive rate
Case
Control

Figure S2. The ROC curve and boxplot of an individual biomarker. The sensitivity is on the y-axis, and the specificity is on the x-axis. The area-under-the-curve is in blue. Boxplots show the concentrations of the selected feature between the two groups. A horizontal line is in red, indicating the optimal cutoff.

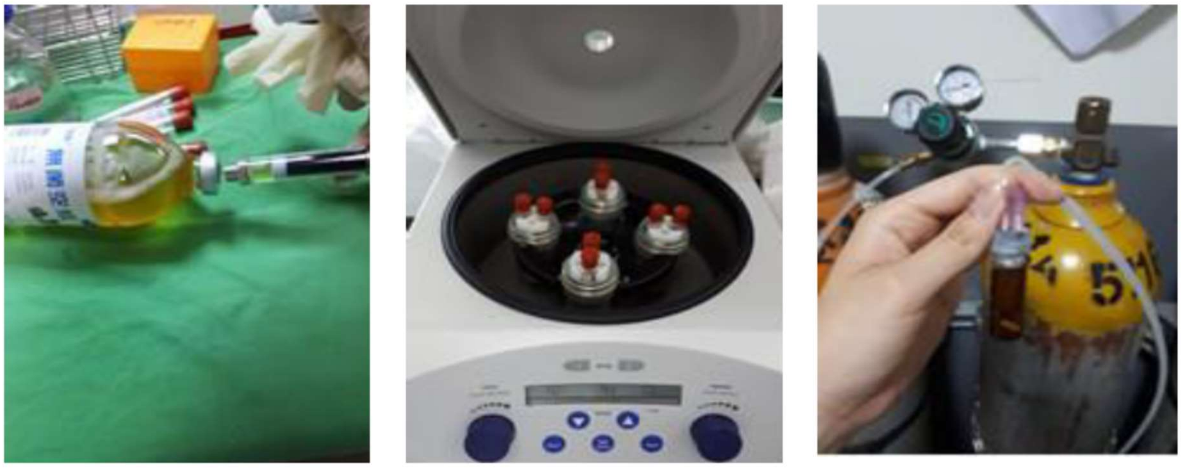

(a) (b) (c)

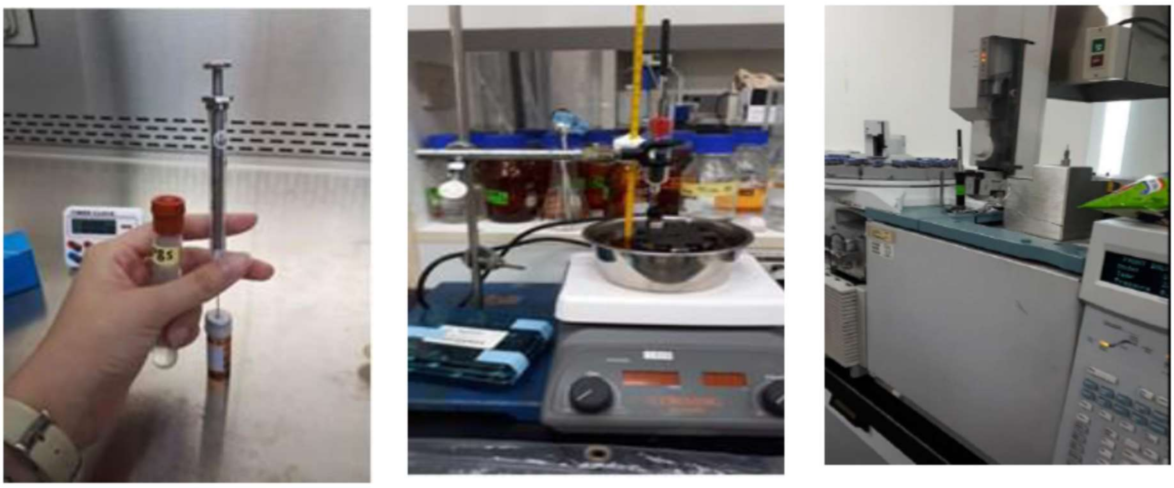

(d) (e) (f)

Figure S3. Standardized procedures for the VOC analysis include: (a) using a gas-tight syringe to collect pleural fluid from a sterile bottle; (b) centrifuging the sample at 1500 x g for 10 min at 4 °C, (c) filling the 4-mL glass vial with nitrogen gas; (d) injecting 2 mL of supernatant into the sealed vial; (e) inserting the SPME fiber into the vial for extraction; and (f) performing GC-MS analysis.

Reference

1 Pluskal, T., Castillo, S., Villar-Briones, A. & Oresic, M. MZmine 2: modular framework for processing, visualizing, and analyzing mass spectrometry-based molecular profile data. *BMC Bioinformatics* **11**, 395, doi:10.1186/1471-2105-11-395 (2010).

2 Jiang, Y., Zhao, L., Yuan, M. & Fu, A. Identification and changes of different volatile compounds in meat of crucian carp under short-term starvation by GC-MS coupled with HS-SPME. *Journal of Food Biochemistry* **41**, doi:10.1111/jfbc.12375 (2017).

3 Hayashi, S. *et al.* A novel application of metabolomics in vertebrate development. *Biochem Biophys Res Commun* **386**, 268-272, doi:10.1016/j.bbrc.2009.06.041 (2009).

4 Niu, W., Knight, E., Xia, Q. & McGarvey, B. D. Comparative evaluation of eight software programs for alignment of gas chromatography-mass spectrometry chromatograms in metabolomics experiments. *J Chromatogr A* **1374**, 199-206, doi:10.1016/j.chroma.2014.11.005 (2014).

5 Xia, J. G. & Wishart, D. S. Web-based inference of biological patterns, functions and pathways from metabolomic data using MetaboAnalyst. *Nature Protocols* **6**, 743-760, doi:10.1038/nprot.2011.319 (2011).
